# Asymmetric Dimethylarginine and Hepatic Encephalopathy: Cause, Effect or Association?

**DOI:** 10.1007/s11064-016-2111-x

**Published:** 2016-11-25

**Authors:** Anna Czarnecka, Krzysztof Milewski, Magdalena Zielińska

**Affiliations:** Department of Neurotoxicology, Mossakowski Medical Research Centre, Polish Academy of Sciences, 5 Pawińskiego Street, 02-106 Warsaw, Poland

**Keywords:** Hepatic encephalopathy, Asymmetric dimethylarginine, l-Arginine, Nitric oxide synthase, Cerebral blood flow

## Abstract

The methylated derivative of l-arginine, asymmetric dimethylarginine (ADMA) is synthesized in different mammalian tissues including the brain. ADMA acts as an endogenous, nonselective, competitive inhibitor of all three isoforms of nitric oxide synthase (NOS) and may limit l-arginine supply from the plasma to the enzyme via reducing its transport by cationic amino acid transporters. Hepatic encephalopathy (HE) is a relatively frequently diagnosed complex neuropsychiatric syndrome associated with acute or chronic liver failure, characterized by symptoms linked with impaired brain function leading to neurological disabilities. The l-arginine—nitric oxide (NO) pathway is crucially involved in the pathomechanism of HE via modulating important cerebral processes that are thought to contribute to the major HE symptoms. Specifically, activation of this pathway in acute HE leads to an increase in NO production and free radical formation, thus, contributing to astrocytic swelling and cerebral edema. Moreover, the NO-cGMP pathway seems to be involved in cerebral blood flow (CBF) regulation, altered in HE. For this reason, depressed NO-cGMP signaling accompanying chronic HE and ensuing cGMP deficit contributes to the cognitive and motor failure. However, it should be remembered that ADMA, a relatively little known element limiting NO synthesis in HE, may also influence the NO-cGMP pathway regulation. In this review, we will discuss the contribution of ADMA to the regulation of the NO-cGMP pathway in the brain, correlation of ADMA level with CBF and cognitive alterations observed during HE progression in patients and/or animal models of HE.

## Hepatic Encephalopathy

Hepatic encephalopathy (HE) is a complex neuropsychiatric disorder that results from impaired liver function, i.e. insufficient clearance of toxins from blood, which in excess enter the brain. The impaired liver function results from acute or chronic liver failure (ALF vs. CLF) and is associated with a wide range of neurological alterations, including cognitive and motor disturbances mainly accompanying CLF [1]. A rapid progress of HE due to ALF, leads to cerebral edema and increased intracranial pressure followed by cerebral herniation and death [2].

The cellular and molecular mechanisms underlying HE are extremely complex and have not been elucidated enough, yet. However, there is a consensus that HE is mainly associated with an interference of ammonia with various aspects of brain metabolism, leading to imbalance of neural transmission [3–5]. HE is also named a primary “astrogliopathy”, because ammonia affects astrocytes, housekeepers of the central nervous system, thus impairing astrocyte-neuronal interactions, and contributing to neurotransmitter imbalance.

Dysregulation of nitric oxide (NO) production and subsequent derangement of guanidine triphosphate conversion to cyclic guanidine monophosphate (cGMP) [6, 7] is a common denominator of most of the symptoms accompanying ALF and CLF progression. At low nM concentrations, NO is an important intracellular messenger that activates soluble guanylate cyclase (sGC), initiating the cGMP production. In acute HE, ammonia-induced over-stimulation of ionotropic (mainly NMDA) glutamate receptors and activation of nitric oxide synthase (NOS) leads to an increase in NO synthesis further contributing in the generation of reactive oxygen and nitrogen species (ROS/RNS) in the brain [8–11]. On the other hand, decreased cGMP signaling in the brain has been identified as a key cause of cognitive dysfunction and memory impairment associated with chronic HE [12].

## Asymmetric Dimethyl l-Arginine (ADMA), an Endogenous Nitric Oxide Synthase Inhibitor

In 1992 asymmetric (N^G^, N^G^) dimethylarginine (ADMA) was first described as an endogenous inhibitor of NOSs [13]. ADMA, its symmetric isoform (N^G^, N^G^) dimethylarginine (SDMA) and N^G^-monomethyl-l-arginine (monomethylarginine; l-NMMA) can regulate NO synthesis by inhibiting NOS and/or can compete for cationic amino acid transporters, which supply NOS with l-arginine [14]. ADMA is a pan-inhibitor of all three NOS isoforms, being a potent noncompetitive inhibitor of neuronal and endothelial NOS and a week inhibitor of inducible NOS. All methylated derivatives of l-arginine are ubiquitous in mammalian cells, exported from their site of origin, and imported from the plasma at distant sites by cationic amino acid transporters in exchange for l-arginine and other cationic amino acids [14, 15]. Since their discovery, the role of these compounds in the regulation of NO production has attracted increasing attention. Interestingly, next to its association with cardiovascular disease, ADMA seems also to play a role in other clinical conditions, such as critical illness, diabetes mellitus, kidney failure and hepatic failure [16, 17]. Although circulating l-arginine levels may be >100 times higher than those of ADMA, recent investigations have shown that in peripheral endothelial cells (a) intracellular ADMA: l-arginine ratio (an index of NO bioavailability) is significantly higher than the ratio measured in plasma and (b) significant NOS inhibition is achieved at physiological levels of endogenous methylarginines [18]. Faraci et al. [19] found that 50% of rat brain NOS activity was inhibited by infusion of ADMA even at low or physiological ADMA concentrations [19]. It is now well established in vitro and in vivo that micromolar concentrations of ADMA and l-NMMA, can compete with l-arginine for cell membrane transport sites. Considering that human body generates approximately 300 μmol (approximately 60 mg) of ADMA per day [14], which results in plasma ADMA concentration between 0.4 and 0.7 µM [20], and that ADMA is mainly released from myelin basic proteins highly expressed in neuronal tissue, the above evidences suggest that endogenous methylarginines may contribute to the regulation of NO levels.

### ADMA Metabolism

Free methylated arginine derivatives are formed endogenously by the sequential processes of protein methylation and proteolysis by intracellular proteases and/or the proteasomal system [21]. The methylation of protein arginine residues is catalyzed by protein-methyl transferase (PRMT) family enzymes of which at least 11 mammalian isoforms have been described [22]. PRMT-1 is the main ADMA-generating enzyme. There are two known metabolic pathways for the removal of ADMA in mammals: (1) hydrolysis of ADMA to citrulline and dimethylamine in the cytoplasm by dimethylarginine dimethylaminohydrolases (DDAH-1 and DDAH-2) and (2) transamination of ADMA to α-keto-δ-(*N,N*-dimethylguanidino) valeric acid (DMGV) by alanine-glyoxylate aminotransferase 2 (AGXT-2) [23]. The role of the kidney and the liver in the metabolism of ADMA has been extensively studied and both organs have been proven to play a key role in the elimination of ADMA. The liver removes the majority (~80%) of ADMA exclusively via its degradation by DDAH, while the kidney uses both metabolic degradation by DDAH and urinary excretion to eliminate ADMA. DDAHs co-localize with different NOS isoforms [24], providing further indirect evidence that these enzymes may be involved in controlling the local availability of NO and downstream responses. DDAH-1 is highly expressed in the brain, suggesting its specific function in this organ. The coexistence of neuronal NOS (nNOS) and DDAH-1 in brain tissues suggests that ADMA may play some special role in the central nervous system and may be more than just an inert metabolic product. Inhibition of DDAH leads to an increase in ADMA levels and thus to a decrease in NO production. Since this pathway is regulated by complex feedback mechanisms, it probably has the ability to act as a stop signal for excessive NO production, thus potentially curbing its pathogenic action, while leaving physiological NO functions intact. Much less is known about the physiological role of AGXT-2 in ADMA metabolism. AGXT-2 is a pyridoxal phosphate-dependent aminotransferase that, in the rat, is expressed at high levels in the kidney [25] and brain [26]. AGXT-2 can also utilize ADMA as a donor of amino groups, leading to the formation of DMGV [27–29]. In this context, down-regulation of DDAH could result in an increased contribution of AGXT-2 to the metabolism of ADMA in pathophysiological conditions.

## ADMA in Liver Dysfunction: Implications to the HE

A growing body of data suggests that increased concentration of ADMA, which is relatively stable and can be accurately measured in the plasma, accompanies liver dysfunctions in a wide sense and HE (for consolidated data see Table 1).

**Table 1:** The summary of recent studies on asymmetric dimethylarginine (ADMA) in acute and chronic liver failure in patients and animal models of HE

Reference	Subjects	Localization	ADMA	l-Arg	l-Arg/ADMA	DDAH	Findings
Bajaj et al. [30]	Patients, (CLF) cirrhosis, TIPS	Plasma	Increased	–	–	–	ADMA levels higher in patients with HE, correlation with cognitive dysfunction
Brenner et al. [31]	Patients, (ALF)/sepsis	Plasma	Increased	Increased/decreased	Decreased	–	Measurements of ADMA and l-arg at sepsis onset appeared to be early predictors for survival in septic patients with ALF
Mookerjee et al. [32]	Patients, (ALF) (acetaminophen)	Plasma	Increased	–	–	–	Correlation between ADMA levels and proinflammatory cytokines
Mookerjee et al. [33]	Patients, (CLF) alcoholic hepatitis	Plasma, liver	Increased in the plasma and liver	–	–	Decreased in the liver (DDAH2)	Alcoholic hepatitis patients have higher portal pressures associated with increased ADMA
Lluch et al. [34]	Patients, (CLF) alcoholic cirrhosis	Plasma	Increased	–	Decreased	–	ADMA might oppose the peripheral vasodilation caused by excessive NO production in severe cirrhosis
Vizzutti et al. [35]	Patients, (CLF) hepatitis C virus	Plasma	Increased	–	–	–	ADMA correlates with portal pressure
Nijveldt et al. [36]	Patients (major hepatectomy)	Plasma	Increased	–	–	–	Increased levels of ADMA occur in the postoperative course after a major hepatic resection
Milewski et al. [37]	Rat, TAA (ALF)	Plasma, brain	Increased	–	Decreased	Decreased activity	Histidine decreased brain ADMA level
Bekpinar et al. [38]	Rat, TAA (ALF)	Plasma, liver	Increased in the plasma	Decreased in the plasma	Decreased in the plasma	Decreased activity in the liver	Rosiglitazone improved the plasma arginine/ADMA ratio
Develi-Is et al. [39]	Rat, TAA (ALF)	Plasma, liver	Increased in the plasma	Decreased in the plasma	–	Decreased activity in the liver	Hemin increased DDAH activity
Bal et al. [40]	Rat, (LPS)/d-galactosamine (ALF)	Liver	Increased	Increased	No changes	Decreased activity in the liver	Metformin decreased tissue ADMA level and restored the DDAH activity
Ferrigno et al. [41]	Rat, BDL^a^	Liver	Increased	–	–	No changes	Tissue ADMA increases with a CAT-2-dependent mechanism
Sharma et al. [42]	Pig, PCS (ALF)	Plasma	Increased	Decreased	Decreased	–	Relative reduction in l-arginine concentration despite increased de novo production reduction the effect of arginase on NOS by the increase in the levels of ADMA
Balasubramaniyan et al. [43]	Rat, BDL (CLF)	Plasma, brain	Increased	No changes in the brain	Decreased in the brain	Decreased in the brain (DDAH1)	Reduction in ammonia with OP reduces neuroinflammation and restores eNOS activity
Huang et al. [44]	Rat, BDL (CLF) BDL + LPS^b^	Plasma, liver, kidney	Increased in the plasma	–	Decreased in the plasma	–	In cirrhosis with sepsis, simultaneous lowering of ADMA levels and enhancement of l-arginine levels may be an optimal strategy for the treatment of kidney injury
Yang et al. [45]	Rat, BDL (CLF)	Liver	Increased	–	–	Decreased (both isoforms)	Vitamin E suppressed hepatic ADMA and oxidative stress
Huang et al. [46]	Rat, BDL (CLF)	Plasma, brain, liver	Increased in the plasma	No changes in the plasma	Decreased in the plasma	No changes in the brain and the liver	Plasma ADMA plays a role in BDL-induced spatial deficit
Laleman et al. [47]	Rat, TAA (CLF), BDE (CLF) PPVL (CLF)	Plasma	Increased in plasma of BDE rats, no changes in the plasma in TAA rats	–	–	–	In rats with biliary cirrhosis, ADMA may mediate decreased NOS activity

Animal models were ordered according to guidelines accepted by International Society for Hepatic Encephalopathy and nitrogen metabolism [48]

^a^3-Day cholestasis

^b^14-Day cirrhosis with superimposed sepsis

Elevated plasma concentrations of ADMA are observed in patients with severe acute alcoholic hepatitis [33] and acute liver failure [32]. In patients with compensated alcoholic or hepatitis C virus related chronic liver diseases, increased peripheral ADMA have been also reported [34, 35]. Recent data confirmed this observation in a wide cohort of cirrhotic patients [30], likewise in patients with transjugular intrahepatic portosystemic shunt (TIPS) [49].

Studies on the thioacetamide (TAA)-induced rat model of ALF revealed ADMA elevation in the plasma and both in the brain cortex tissue and extracellular space with parallel lowering of liver DDAH activity [37, 38, 50]. In addition, in the BDL rat model, ADMA level significantly raises in the peripheral blood, whereas the concentration of l-arginine decreases [51]. Of note in this context, the PRMT-1 protein content was elevated in the liver of BDL rats [52, 53], but reduced in BDL rat brain [46]. There is a consensus that essential cause of ADMA elevation during liver failure is related to the lowered DDAH activity in the liver which may or may not be in line with lowered DDAH protein expression [29, 43, 54]. Recent data have revealed that DDAH-1 is predominantly present in the parenchymal liver hepatocytes while loss of protein is seen during liver fibrosis in cirrhotic patients, BDL rats and CCl_4_ treated rats [55].

Whether elevated ADMA concentration in the plasma can be considered a potent clinical marker of liver dysfunction and/or an accompanying factor in HE diagnosis, still remains an open question. Nevertheless, even more interesting are possible cerebral and/or systemic consequences of elevated ADMA. As already mentioned, HE is a very complex syndrome in which ADMA may exert its action in different ways, for instance by influencing vascular constriction leading to the CBF regulation, oxidative stress, cognitive function and inflammation. The authors of this review are aware that the presented list must stay “open” due to possible alternative approaches to ADMA function and ambiguously defined pathophysiological processes.

### ADMA and Endothelial Function: A Contribution to the CBF Regulation

Cerebral blood flow (CBF) reflects brain energy demand and as such may be used as a potential indicator of an early decrease in brain activity. A global decrease in brain energy metabolism is one of the primary events associated with the pathogenesis of HE. Reduced cerebral oxygen consumption and CBF was observed in cirrhotic patients with an acute episode of overt HE, but not in cirrhotic patients without HE [56]. The increased CBF in cortical regions could be a common effect of the TIPS procedure, while decreased global CBF following TIPS might indicate the development of overt HE [57, 58]. Additionally, a pronounced decrease in the CBF in the cerebral cortex and whole brain was demonstrated in our laboratory in the rat TAA model of ALF [unpublished data, 59]. On the other hand, the increased CBF was reported to correlate with raised intracranial pressure and inflammatory markers in patients with ALF [60]. In general, the values of CBF reported in ALF are variable. A high CBF was demonstrated in patients with ALF in the late stage of the disease but before the development of cerebral herniation [61]. Contrary, Almdal et al. [62] reported low CBF in patients in more advanced stages of HE [62]. A study in 30 patients in various stages of HE suggested that the CBF was likely to be low [63]. Simultaneous measurement of ICP and CBF in eight patients revealed that ICP >24 mmHg was correlated with high CBF [64]. Felipo [2] in his comprehensive review presented the hypothesis that CBF was differently regulated in the cerebral cortex and cerebellum as well as at the early and late stages of HE [2]. However, this assumption is not entirely consistent with all the available data presented above.

The restriction of CBF may be one of effects of ADMA. NO is arguably the most important endogenous vasodilator regulating the perfusion of the brain, significantly influencing the tone of conductive and resistance arteries as well as venous vessels [65]. Exogenous ADMA causes concentration- and endothelium-dependent contractions of the human middle cerebral artery [66]. Similar study was conducted on rings of human middle cerebral artery from 26 autopsies, where the effects of exogenously administered ADMA were prevented by l-arginine [67]. On the one hand, ADMA might contribute to brain injury by reduction of CBF while on the other, ADMA might be involved in NOS-induced oxidative stress and excitotoxic neuronal death. After ischemic stroke, the inhibition of inducible NOS (iNOS) and nNOS have been suggested to be neuroprotective while eNOS inhibition might reduce CBF after brain injury [68]. Taken together, the effects of ADMA, which acts as a nonselective NOS inhibitor and a mediator of oxidative stress via uncoupling of iNOS and eNOS, may be multifarious, either detrimental or beneficial. The explanation of this issue requires further studies.

### ADMA and Oxidative-Nitrosative Stress

A growing body of evidence suggests that methylated derivatives of l-arginine can regulate NOS-derived superoxide production by an uncoupled nNOS [69] or eNOS [70]. Oxygen species can oxidize tetrahydrobiopterin (BH_4_) to dihydro-(BH_2_), which uncouples eNOS. Since ROS may increase intracellular ADMA levels, this is a potential positive feedback mechanism to perpetuate oxidative stress [71]. However, the effects of ADMA on nNOS are different from eNOS. In the presence of BH_4_, superoxide production by nNOS was independently inhibited by both ADMA and l-arginine, whereas neither ADMA nor l-arginine altered superoxide formation by eNOS in the absence of BH_4_ [69]. It was also reported that ADMA adduction to murine epithelial cells induced rapid increases in superoxide production, inhibited NO synthesis, and caused peroxynitrite formation. These effects of ADMA were exerted via uncoupling of iNOS [72].

Considering this, it is tempting to speculate that the observed induction of oxidative stress in HE may be modulated by ADMA. In vivo evidence for ammonia-induced oxidative stress in the brain has been obtained in animal models of acute ammonia intoxication [11, 73] and in cultured astrocytes acutely exposed to ammonia in vitro [74]. Recent works also documented an induction of oxidative stress in cirrhotic rats mainly via an overproduction of superoxide associated with a significant reduction in NO bioavailability accompanying the increased levels of nitrosylated proteins [75]. Oxidative stress may directly modulate ADMA level via its impact on ADMA metabolizing enzymes. In BDL rats, elevation in plasma and hepatic ADMA levels were positively correlated with disease severity and oxidative stress markers [52]. Also, both PRMT-1 protein expression and oxidative stress markers were elevated in the liver of this model [52]. However, a study on hepatocytes did not confirm an association of PRMT-1 expression and ROS activation [54].

Previous works indicated DDAH sensitivity to oxidative stress [76, 77]. The proposed mechanism of the inhibition of DDAH activity was associated with imbalanced pro-oxidant/antioxidant state of sulfhydryl groups in the active site of the enzyme. Indeed, the expression and activity of DDAH in hepatocytes in vitro were suppressed by superoxide and H_2_O_2_ in a time-dependent manner [54]. This assumption has been confirmed by reduction of the increased ADMA level and restoration of DDAH activity after administration of compounds with antioxidant properties, such as melatonin [52], l-histidine [37] or vitamin E which suppressed hepatic ADMA level and oxidative stress determined in the hepatic circulation in the rat BDL model [53].

### ADMA and Cognitive Impairment

Manifestations of intellectual dysfunction in HE patients include psychomotor slowing, impaired attention and reduced ability to perform calculations [78, 79]. As HE worsens, impairment of speech and orientation, followed by temporal and spatial disorientations appears [80]. The most comprehensive research of Bajaj et al. [30], based on various cognitive tests, reported the association of ADMA concentration with cognitive dysfunction and inflammation in cirrhosis independently of the severity of liver disease [30]. Moreover, those authors showed that ADMA levels were significantly higher in patients who developed HE after TIPS placement compared to those who remained free of HE [30]. Memory impairment was also widely described in rats with CLF [12, 81, 82]. Furthermore, there are data indicating that the glutamate-NO-cGMP pathway in the cerebellum modulates some of types of learning, particularly the ability to learn a Y maze task [2]. Therefore, the brain ADMA and its related enzymes, involved in endogenous NO production, can be a potential cause of these disturbances. Interestingly, ADMA may contribute to brain dysfunction in patients with Alzheimer’s disease and stroke [83, 84]. Elevated peripheral ADMA may play a role in spatial deficit in BDL rats. However, authors of that study also found increased plasma ADMA levels in one of the studied groups of rats without accompanying cognition impairment [23]. On the other hand, spatial memory alterations were also observed in portacaval shunt (PCS), portal hypertension and chronic TAA intoxication models in which ADMA elevation was not precisely confirmed [85].

With a high probability, cognitive deficits in HE and chronic liver disease are linked to changes in CBF [86, 87]. It is also possible that high ADMA levels are likely to uncouple eNOS, leading to superoxide generation [34, 43] and may provide an additional mechanism leading to the worsened spatial performance.

### ADMA and Inflammation

Systemic inflammation is associated with enhanced plasma ADMA levels and follows endothelial dysfunction in various inflammatory diseases, such as atherosclerosis and rheumatoid arthritis (RA) [88, 89]. Higher levels of methylarginines also correlated with an increase in mortality of patients with sepsis [90]. More recently an important role of inflammation, as an accompanying factor during HE development, has been postulated [91]. Elevated blood levels of pro-inflammatory cytokines [interleukin-1b (IL-1b), interleukin-6 (IL-6), tumor-necrosis factor-alpha (TNFα)] correlate positively with the severity of HE [92–94]. ADMA levels were markedly higher in ALF patients compared to age-matched controls, and better correlated with the levels of pro-inflammatory cytokines in pre-transplantation patients undergoing hepatic venous catheterization. Following liver transplantation, both ADMA levels and pro-inflammatory markers were reduced [32]. Comparison of patients with decompensated alcoholic cirrhosis and acute hepatitis to the patients with alcoholic cirrhosis alone revealed that former ones demonstrated a much higher increase in inflammatory response markers and ADMA blood level. Furthermore, these observations were in line with down-regulation of DDAH-2 protein expression and up-regulation of PRMT-1 protein in the liver [33]. Our group showed in the TAA-induced ALF model an increase in both plasmatic/brain ADMA and TNF-α. Moreover, increase of TNF-α mRNA was observed in the brain cortex [37]. Elevated plasma and brain TNF-α level with accompanying increase of ADMA protein were also described in cirrhosis rats [43].

### ADMA and Suggested Therapeutic Strategies

A few treatment strategies used to cure hypertension, chronic kidney disease, hyperlipidemia or diabetes additionally reduce the increased level of ADMA. These include inhibitors of the renin-angiotensin-aldosterone system [95, 96], statins [97], fibrates and niacin [98, 99] or thiazolidinediones [100]. Also, antioxidants [53] or aspirin [101] contribute to the regulation of abnormal ADMA level in various disorders. So far, homocysteine-lowering therapy, despite a few promising attempts, has not been very successful in reduction of ADMA [95, 102]. The linkage between anti-inflammatory drugs and ADMA lowering therapy was recently reported in RA. Three-week treatment with etanercept or adalimumab reduced in those patients ADMA level in plasma [103]. However, previous study did not reveal an impact of 18-month methotrexate or adalimumab treatment on ADMA serum levels in RA patients [104].

Supplementation of l-arginine has also been suggested to be able to eliminate the negative ADMA impact [105]. Theoretically, in the presence of pathophysiologically relevant concentrations of ADMA and physiological concentration of l-arginine, the eNOS activity decreases which results in the NO formation rates below the physiological level. In such conditions, supplementation with exogenous l-arginine displaces the competitive inhibitor and restores the physiological l-arginine/ADMA ratio [106]. l-Arginine is the principal substrate of NOS and several early studies in human and animal models reported the beneficial effects of acute and chronic l-arginine supplementation on endothelial NO production [107, 108]. However, there are inconsistent results in a clinical context. It was reported that five of 17 published human studies showed no vascular health benefits of oral l-arginine supplementation [109]. Moreover, Wilcken et al. [10] reported that l-arginine affected ADMA metabolism providing a relative stable ADMA/l-arginine ratio despite frequent changes in the plasma level of l-arginine [110]. They concluded that the regulatory role of l-arginine on ADMA might explain the unexpected results in some l-arginine supplementation studies.

Taking into consideration that intracellular ADMA is mainly regulated by PRMT and DDAH, the use of specific PRMT inhibitors or DDAH agonists might be a more reasonable therapeutic strategy. However, due to a high degree of sequence conservation across the PRMT family, creation of specific PRMT inhibitors is challenging [111]. In addition, PRMT enzymes are involved in complex cellular physiology and PRMT inhibition may give rise to side effects. The development of PRMT-1-specific inhibitors is a key objective in the search for more efficient therapeutic strategies. Initial experiments demonstrated that irreversible PRMT inhibition by S-adenosyl-l-homocysteine hydrolase blocks methylation in the cell and has both preventive and therapeutic potential in an animal model of arthritis [112]. It appears that future efficient PRMT inhibitors will rather normalize than completely inhibit the PRMT-1 function, restoring ADMA to normal levels. Since ADMA inhibits NOS activity, this could result in restoration of NO production, overcoming many important secondary effects of diseases.

The primary route of elimination of hepatic ADMA involves its hydrolysis by DDAH-1. The farnesoid X receptor (FXR) belongs to a family of nuclear hormone receptors that have an important role in maintenance of bile, lipid and glucose balance [113]. A synthetic FXR agonist was shown to significantly increase hepatic DDAH-1 gene expression in diabetic rats [114] allowing for the determination of DDAH-1 as an FXR target gene. Subsequently, further studies in rodent models of cirrhosis and hypertension have determined the efficacy of FXR agonist in increasing DDAH-1 expression [55, 115, 116]. DDAH-1 augmentation was associated with a decrease in portal pressure, reduced fibrosis and decreased hepatic ADMA levels. Furthermore, Balasubramaniyan et al. [43] demonstrated that administration of ornithine phenylacetate in the BDL model of chronic liver cirrhosis decreased the abnormal brain ADMA level by restoring DDAH-1 expression concomitantly with reduction of brain ammonia and inflammation [43].

Finally, therapeutic up-regulation of AGXT-2 may have advantages compared with the up-regulation of DDAH-1 or DDAH-2, because the latter two enzymes may exert cancer-promoting effects that are independent of ADMA [117]. Pharmacological approaches aimed to increase the activity of AGXT-2 could have potential therapeutic value in pathological conditions in which ADMA acts as a mediator of pathogenesis.

The question arises which of the above-mentioned therapeutic strategies could be beneficial in treatment of hepatic encephalopathy? Some doubts have been raised as to whether 10–24% decreases in plasma ADMA levels induced by these agents in different diseases can be beneficial. Furthermore, the increase in ADMA level in most diseases (except for renal failure and severe shock) is relatively minor and it is unclear if this is sufficient to induce a significant NOS blockade. However, any potential strategy able to lower high plasma ADMA levels should be considered beneficial in the therapy of HE patients.

## Summary and Perspectives

**Fig. 1:**
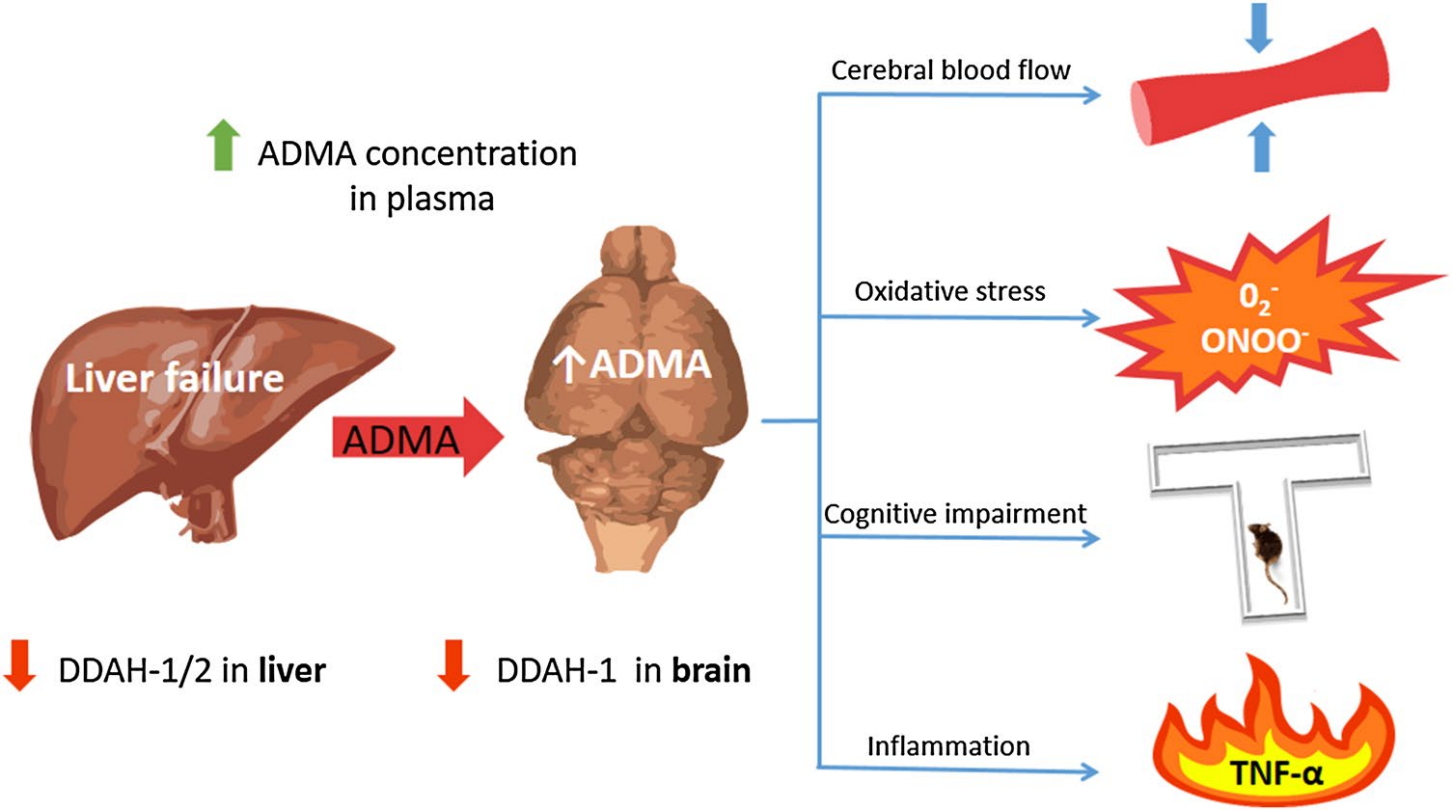
A potential contribution of the elevated ADMA level to the cerebral impairment occurring in the HE. Acute or chronic liver failure results in the increased level of ADMA in peripheral tissues and in the brain, due to its decreased degradation by the enzyme DDAH, among other things. High level of ADMA contributes to the restriction of the cerebral blood flow, oxidative stress, cognitive impairment and inflammation

The molecular background underlying HE is still not completely understood and current treatment is rather symptomatic than mechanism-based. The observations that elevated ADMA levels predict future outcomes in cohort studies associated with cardiovascular diseases demonstrated the potential for methylarginines to act as a marker also in liver failure accompanying HE pathology. To date only circumstantial and correlative evidences for the role of ADMA as a mediator of selected processes in HE are available (Fig. 1). The increased circulating ADMA levels may be associated primarily with endothelial dysfunction that somehow can be translated into changes in CBF considered as a causative and/or predictive factor of overt HE. However, the exact mechanism, by which direct effects of ADMA in the brain are translated into CBF changes during HE has not been elucidated in detail. Next, a direct link between increased plasma ADMA concentration and cognitive impairment cannot be definitely confirmed due to a limited number of reports and correlative assumption. Formation of NO is regulated by both l-arginine availability and the presence of the NOS inhibitor ADMA, which may be represented by their ratio (l-arginine/ADMA). However, the application of the l-arginine/ADMA ratio is much limited due to the fact that l-arginine levels vary in a wider range than ADMA levels in the circulation, and, therefore, the ratio needs not reflect the intracellular situation. ADMA appears to regulate the cellular tissue level of NO and, thus, its biological impact both by inhibiting NO production and enhancing NO bio-inactivation by ROS. The primary role of NO synthesis in the pathogenesis of HE, plus a degree of tissue/cell specificity of the enzymes controlling methylarginine levels suggest that the modulation of ADMA metabolism may be considered also as a potential target for future therapeutic interventions. However, the modulation of DDAH and/or AGXT-2 activity and/or expression is still under research. Elucidation of the significance of ADMA in HE will require a significant broadening of the scope of research.

## Abbreviations

ALF: Acute liver failure
ADMA: Asymmetric dimethylarginine
BH_4_: Tetrahydrobiopterin
BDE: Bile duct excision
BDL: Bile duct ligation
CAT: Cationic amino acid transporter
CBF: Cerebral blood flow
CLF: Chronic liver failure
cGMP: Cyclic guanosine monophosphate
DDAH: Dimethylarginine dimethylaminohydrolase
eNOS: Endothelial NOS
HE: Hepatic encephalopathy
iNOS: Inducible NOS
nNOS: Neuronal NOS
NO: Nitric oxide
NOS: Nitric oxide synthase
PCS: Portacaval shunt
PPVL: Partial portal vein ligation
PRMT: Protein arginine methyltransferase
SDMA: Symmetric dimethylarginine
TAA: Thioacetamide
TIPS: Transjugular intrahepatic portosystemic shunt
